# Executive functioning of patients with congenital heart disease: 45 years after surgery

**DOI:** 10.1007/s00392-023-02187-3

**Published:** 2023-04-09

**Authors:** C. Pelosi, R. M. Kauling, J. A. A. E. Cuypers, E. M. W. J. Utens, A. E. van den Bosch, I. Kardys, A. J. J. C. Bogers, W. A. Helbing, J. W. Roos-Hesselink, Jeroen S. Legerstee

**Affiliations:** 1 Department of Cardiology, Erasmus MC, Rotterdam, The Netherlands; 2 Academic Center for Child Psychiatry Levvel, Amsterdam UMC, University of Amsterdam, Amsterdam, The Netherlands; 3 Department of Child and Adolescent Psychiatry/Psychology, Erasmus Medical Center-Sophia Children’s Hospital, Rotterdam, The Netherlands; 4 Department of Cardiothoracic Surgery, Erasmus MC, Rotterdam, The Netherlands; 5 Division of Cardiology, Department of Pediatrics, Erasmus University Medical Center, Sophia Children’s Hospital, Rotterdam, The Netherlands

**Keywords:** Congenital heart disease, Executive function, Psychology, Long-term follow-up

## Abstract

**Background:**

Nowadays, more than 90% of patients with congenital heart disease (CHD) reach adulthood. However, long-term impact on neurodevelopment and executive functioning in adults with CHD are not completely understood.

**Purpose:**

To investigate the self- and informant-reported executive functioning in adults with CHD operated in childhood.

**Material and methods:**

Longitudinal study of a cohort of patients (*n* = 194, median age: 49.9 [46.1–53.8]) who were operated in childhood (< 15 years old) between 1968 and 1980 (median follow-up time: 45 [40–53] years) for one of the following diagnoses: atrial septal defect (ASD), ventricular septal defect (VSD), pulmonary stenosis (PS), tetralogy of Fallot (ToF) or transposition of the great arteries (TGA). Behavior Rating Inventory of Executive Function-Adult version (BRIEF-A) questionnaire was used to assess self- and informant-reported executive functioning.

**Results:**

40–53 years after surgery, the CHD group did show significantly better executive functioning compared to the norm data. No significant difference was found between mild CHD (ASD, VSD and PS) and moderate/severe CHD (ToF and TGA). Higher education, NYHA class 1 and better exercise capacity were associated with better self-reported executive functioning, whereas females or patients taking psychiatric or cardiac medications reported worse executive functioning.

**Conclusions:**

Our findings suggest favorable outcomes (comparable to normative data) regarding executive functioning in adults with CHD, both self- and informant-reported. However, further study is warranted to explore more in detail the different cognitive domains of executive functioning in these patients.

**Graphical abstract:**

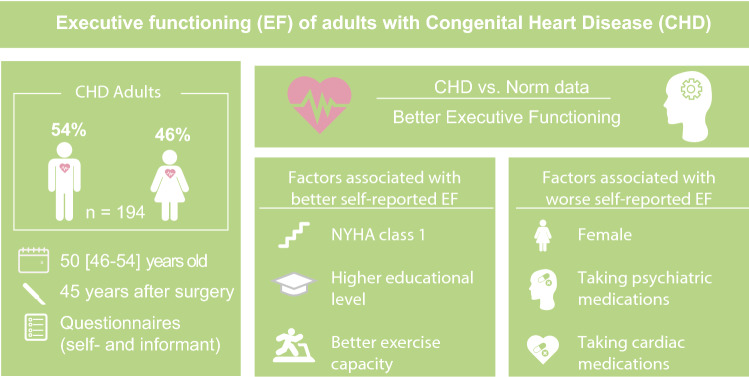

**Supplementary Information:**

The online version contains supplementary material available at 10.1007/s00392-023-02187-3.

## Introduction

Congenital heart diseases (CHD) affect almost 1% of all newborns [1]. Thanks to the dramatic improvements in diagnostic, surgical, and medical care, the survival of these patients dramatically improved nowadays and the prevalence of adults with CHD is 3000 per million adults [2]. However, it was shown that newborns and children with CHD are exposed to a higher risk of neurodevelopmental impairment [3, 4]. Prenatally, the inadequate delivery of oxygen to the brain seems to play a fundamental role; whereas, postnatally, the hemodynamic instability is an important factor which may affect the normal development of the brain in newborns with CHD [3]. In addition, neurodevelopment in these children is supposed to be influenced by multiple perioperative factors such as hemodilution, tissue oxygenation changes, and non-pulsatile blood flow from hypothermic low-perfusion due to circulatory arrest [5].

The impact of CHD on the neurological development is already clear prenatally; therefore, many infants requiring treatment present neurodevelopmental impairment already before cardiac surgery [6, 7]. Furthermore, the neurodevelopmental impairment remains in adolescents with CHD and particularly in those with cyanotic lesions [8]. Specifically, deficits in flexibility/problem solving and verbally mediated executive functioning (EF) were reported [6, 8, 9]. EF encompasses a cluster of skills involved in higher-order and goal-directed behaviors associated with cognitive and behavioral components. They encompass, for instance, the ability to inhibit not suitable behaviors, to shift between different activities, to control emotions, to focus attention or to plan and organize [10]. All these functions are necessary to learn new skills, remember them and use them to solve everyday problems, which are fundamental to live and work independently.

Only a few studies have focused on neurodevelopmental outcomes and EF in adults with CHD. These studies were characterized by heterogeneity of assessment tools and by diversity of CHD severity. The results of these studies were contradictory. Whereas some studies showed no differences between CHD and general population in terms of EF, others demonstrated executive dysfunction [11–16].

The aim of this study was to investigate self- and informant-reported EF in 194 patients with CHD, aged between 40 and 66, and operated in the Erasmus Medical Center (MC), Rotterdam, the Netherlands, between 1968 and 1980. Based on previous studies, we expected worse EF outcomes of the CHD cohort when compared to the normal Dutch population. Furthermore, we aimed to identify possible risk factors, such as the current cardiac health, which could predict EF impairment in adults with CHD. For this purpose, patients were followed every 10 years, and for the current investigation we used our long-term follow-up data (median follow-up time: 45 [40–53] years) [17–23].

## Methods

### Patient sample

This study is part of a multidisciplinary longitudinal study following a large cohort of consecutive patients operated for CHD at young age. Our original cohort consisted of 597 consecutive non-syndromic patients diagnosed with atrial septal defect (ASD), ventricular septal defect (VSD), pulmonary stenosis (PS), tetralogy of Fallot (ToF) or transposition of the great arteries (TGA), who underwent cardiothoracic surgery at young age (< 15 years) at Erasmus MC (Rotterdam, the Netherlands), between 1968 and 1980. This cohort has been investigated every 10 years (1991, 2001, 2011 and 2021). The target population of the current follow-up study consisted of 431 patients who were alive at the time of enrollment. Of these group, 343 patients, who were traceable and who participated at least at two of the previous follow-up studies, received an invitation to participate to this follow-up (Table 1S—supplementary material) . Patients were classified into two groups according to the classification of adults with CHD according to the European Society of Cardiology: mild CHD (*n* = 131), which encompass ASD, VSD, and PS and moderate/severe CHD (*n* = 63), including corrected ToF and TGA after Mustard procedure [24].

### Assessment procedure

The research protocol was approved a priori by the local institutional ethical committee (MEC-2019 0465) and followed the ethical guidelines of the 1975 Declaration of Helsinki. All patients were approached uniformly and before participating in the study, they signed an informed consent. Patients were invited to visit our outpatient clinic between 2020 and 2022 where they underwent psychological questionnaires and in-depth cardiac examinations including echocardiography, ECG, exercise test, cardiac visit, cardiac magnetic resonance or, when not possible, computer tomography, 24-h Holter registration, and blood tests. Before their visit at the hospital, the patients received questionnaires for the psychosocial assessment via email and they completed them digitally at home via a privacy protected website (GemsTracker, Copyright©2011, ErasmusMC and Equipe Healthcare companies). Due to delays in producing the digital questionnaires or due to personal reasons (e.g., no access to email, difficulties to reach the hospital), 37 patients and 48 informants completed the paper version.

### Instruments

*EF assessment* In this study, we used the Behavior Rating Inventory of Executive Function-Adult version (BRIEF-A) questionnaire to assess self- and informant-reported EF of CHD adults in their daily activities [25, 26]. This questionnaire is a standardized 75-items scale and the response format is 1 = never, 2 = sometimes, 3 = often. The items are focused on activities of everyday living and they are divided into nine scales which are non-overlapping: Inhibit, Shift, Emotional Control, Self-monitor, Initiate, Working Memory, Plan/Organize, Task Monitor, and Organization of Materials. The sum of the subscales permits to calculate two indexes: the Behavioral Index (BRI) (Inhibit, Shift, Emotional Control, and Self-monitor) and the Metacognition Index (MI) (Initiate, Working Memory, Plan/Organize, Task Monitor, and Organization of Materials). A global executive composite (GEC) score is derived from the sum of all the subscales. Raw scores or converted T-scores based on the Dutch/Flemish population were used. T-scores are standardized scores with mean of 50 and standard deviation (SD) of 10. Higher scores represent worse EF. A T-score higher than 65 is considered clinically relevant.

*Education level* Patients were asked for the highest education level that they achieved through a questionnaire [18, 20]. The answers were classified according to the SOI 2021 (Dutch Standard Classification of Education) of the CBS (Dutch Institute of Statistics) [27].

*Exercise capacity* Maximal exercise capacity was assessed by bicycle ergometer. A 20 Watt increase per minute protocol was used. Maximal exercise capacity is presented as a percentage of the target of healthy adults corrected per age, gender, weight and height.

### Statistical analysis

Baseline medical characteristics of participants and non-participants were illustrated with descriptive statistics: continuous data were presented as mean ± SD, categorical data as percentages. In case of non-normal distribution, median and 25th–75th percentile were presented. Differences between diagnostic groups were analyzed with *χ*^2^ or Fisher’s exact test for categorical variables as appropriate. Independent samples *t* test or Mann–Whitney-*U* test were used for continuous data according to variable distribution (normal or non-normal).

One sample *t* tests were used to compare the *T* test scores of self and informant reports of EF between adults with CHD and normative data of the same age and gender. Effect sizes were calculated using the Cohen’s *d* test. A Bonferroni correction was used to account for the assessment of 36 comparisons, such that *p* < 0.0014 were considered significant. Finally, the percentages of patients in the clinical range were calculated (> 1.5 standard deviation from the norm) [26].

Univariable linear regressions were performed to identify possible biographical and medical predictors of self- and informant-reported EF (i.e., raw scores). The following predictors were tested separately: age, gender, cardiac diagnosis, educational level, duration of pregnancy, weight at birth, palliative surgery before first heart operation, saturation before operation, age at first open-heart operation, aorta clamp and aorta clamp time, re-intervention with cardiopulmonary bypass (CPB), cerebrovascular accidents (CVA) and/or transitory ischemic attack (TIA), heart failure, NT-pro-BNP, maximum exercise capacity, New York Heart Association (NYHA) class, psychiatric medication, cardiac medication, and systemic ventricular function. Predictors with *p* values ≤ 0.20 were entered into a multivariable linear regression model (backward elimination; *p* < 0.05).

Statistical analyses were processed using IBM SPSS Statistics for Windows 28.0 (Armonk, New York, USA).

## Results

### Group characteristics: (Table 1)

**Table 1:** Biological and clinical characteristics of patients with CHD

	Total CHD (*n* = 194)	Mild CHD (*n* = 131)	Moderate/severe CHD (*n* = 63)	*p*
*Biographical status*				
Female	45.9% (89)	48.1% (63)	41.3% (26)	0.372
Age at follow-up (years)	49.9 [46.1–53.8]	50.0 [46.9–54.9]	48.8 [44.4–51.2]	0.003
*Education level* ^a^				
Lower Secondary Higher	31.8% (61) 33.3% (64) 34.9% (67)	28.7% (37) 34.9% (45) 36.4% (47)	38.1% (24) 30.2% (19) 31.7% (20)	0.412
*Medical history*				
Duration of pregnancy (weeks)	40.0 [40.0–40.0]	40.0 [39.25–40.0]	40.0 [40.0–40.0]	0.300
Weight at birth (kg)	3.2 [2.8–3.6]	3.1 [2.7–3.6]	3.3 [2.7–3.6]	0.075
Palliative surgery before the surgical repair	18.6% (36)	3.1% (4)	50.8% (32)	< 0.001
Saturation before operation (%)	94.0 [82.2–97.7]	97.0 [95.0–98.0]	81.0 [74.0–88.5]	< 0.001
*First open-heart surgery*				
Age at first open-heart operation (years)	4.8 [1.3–7.2]	5.4 [2.1–8.4]	2.4 [0.8–5.4]	< 0.001
Clamp of the aorta	93.1% (163)	89.7% (104)	100% (59)**	0.010
Time aorta clamp (min)	39.0 [19.7–52.2]	25.0 [14.2–40.0]	55.0 [46.7–60.0]	< 0.001
*Post-operative course from operation until 2021*				
Re-intervention with CPB	17.5% (34)	6.1% (8)	41.3% (26)	< 0.001
CVA and TIA	4.6% (9)	3.8% (5)	6.3% (4)	0.432
Heart failure	4.1% (8)	0.8% (1)	11.1% (7)	< 0.001
NYHA class 1	86.5% (160)	92.1% (117)	74.1% (43)	0.001
CPET (%)^b^	98.6 ± 21.8	102.2 ± 20.6	89.6 ± 22.3	< 0.001
Cardiac medications	40.7% (79)	32.1% (42)	58.7% (37)	< 0.001
Psychiatric medications (total)^c^	10.8% (21)	10.7% (14)	11.1% (7)	0.929
Anti-depressant	6.7%(13)	7.6% (10)	4.8% (3)	0.454
Minor tranquillizers/anxiolytics	3.1% (6)	2.3% (3)	4.8% (3)	0.652
Other^d^	3.6% (7)	3.1% (4)	4.8% (3)	0.550
NT-pro-BNP	15.5 [9.0–30.2]	14.5 [8.0–24.0]	23.5 [11.0–44.2]	0.009
*Systemic ventricular function*				
Good Reasonable Poor Bad	64.7% (119) 23.9% (44) 9.8% (18) 1.6% (3)	81.0% (102) 15.9% (20) 3.2% (4) 0	29.3% (17) 41.4% (24) 24.1% (14) 5.2% (3)	< 0.001

For continuous variables, median [25th–75th percentile] is reported. For categorical variables, percentage (*n*) is shown. Differences between diagnostic groups were analyzed for the continuous data with Mann–Whitney-*U* test if not normally distributed. *χ*^2^ test was used to analyze differences between categorical data

*CPET* Cardio-pulmonary Exercise Test, *CVA/TIA* cerebrovascular accident/transient ischemic attack, *NYHA* New York Heart Association

^a^Based on SOI 2021 classification

^b^Percentage of expected max exercise capacity for healthy control adjusted per sex, height and age

^c^Some patients use multiple psychiatric medication

^d^Antipsychotic, psychostimulants, and anticonvulsants

Of the invited patients, 194 completed the questionnaire. The informant report was filled out by the patient’s partner (*n* = 161). If this was not possible, it was filled out by someone close to the patient (*n* = 8) (son/daughter, sister-in-law, aunt). In total, 169 informants completed the questionnaire.

The final cohort consisted of 194 patients (45.9% female) aged 49.9 [46.1–53.8] years old. Of the final sample, 34.9% were higher educated. Median age at operation was 4.8 [1.3–7.2] years old, CPB was used in the 93.1% of the surgeries with median time of 39.0 [19.7–52.2] minutes. Since the first surgery, 17.5% of the patients had a re-intervention with CPB. CVA and/or TIA occurred in 4.6% of the patients, while 4.1% had history of heart failure. Cardiac medication was used by 40.7%, and psychiatric medication by 10.8% of the patients. The mean exercise test capacity was 98.6% of the expected and 86.5% of the patients were in NYHA functional class 1, 64.7% had a good systemic ventricular function.

No significant difference was found between participants (*n* = 194) and non-participants (*n* = 237) in terms of diagnosis, sex, and medical characteristics before and at surgery (Table 2S in supplementary material). Non-participant patients were defined as all the alive patients who do not participated in the current follow-up study. Patients with moderate/severe CHD in comparison with mild CHD were younger, received more often palliative surgery, had higher levels of NT-pro-BNP, and had lower oxygen saturation before reparative surgery. In addition, patients with moderate/severe CHD underwent their first open-heart operation at a younger age and with longer aorta clamp time than for the mild CHD. Cardiac medication and re-intervention were more frequent in patients with moderate/severe CHD. Moreover, NYHA functional class was higher, the exercise capacity and systolic function of the systemic ventricle were worse in this group in comparison with mild CHD (Table 1).

### Executive function per age group

**Fig. 1:**
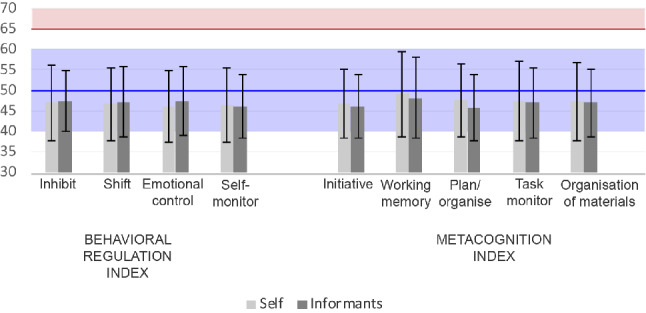
Brief-A self- and informant-reports mean scores per sub-scale reported in T-scores. T-scores are derived from the normative data of the Dutch/Flemish population 18–65 years old [26]. The error bar represents the standard deviation. Higher scores indicate worse executive functioning. The horizontal blue line and area, respectively, represent the mean (T-score = 50) and the standard deviation (SD = 10) of the mean (T-score = 50) of the normal population. The horizontal red line and red area mark represent, respectively, the Brief-A clinical impairment threshold and range (T-score > 65)

For illustrative purposes, Fig. 1 shows the mean T-scores with SD per sub-scale of CHD adults compared to the norm.

The total CHD sample scored significantly lower (better) than the norm population on all indexes and on the GEC for both self (BRI: *t* = − 6.75, *p* < 0.001, MI: *t* = − 4.23, *p* < 0.001, GEC: *t* = − 5.68, *p* < 0.001) and informant reports (BRI: *t* = − 5.97, *p* < 0.001, MI: *t* = − 5.75, *p* < 0.001, GEC: *t* = − 6.35, *p* < 0.001) (Table 2). All the differences remained significant after applying a Bonferroni correction for 36 comparisons.

**Table 2:** Mean BRIEF-A self- and informant-reported raw scores are shown for the CHD cohort and for the norm (total and per age group)

	CHD	Norm	Cohen’s *d*	*t*	95% CI of the difference	*p*
Self-report						
*Total group (n* = *194)*						
BRI	40.6 ± 8.5	44.7 ± 9.6	− *0.48*	− 6.75	[− 5.31; − 2.91]	< 0.001*
MI	54.4 ± 11.6	58.0 ± 12.2	− 0.30	− 4.23	[− 5.17; − 1.88]	< 0.001*
GEC	95.0 ± 18.9	102.7 ± 20.4	− 0.41	− 5.68	[− 10.35; − 5.01]	< 0.001*
*Age group 40–49 (n* = *114)*						
BRI	40.7 ± 8.6	43.9 ± 9.6	− 0.38	− 4.05	[− 4.89; − 1.68]	< 0.001*
MI	54.3 ± 12.2	57.2 ± 12.0	− 0.24	− 2.58	[− 5.20; − 0.69]	0.011
GEC	94.9 ± 19.4	101.2 ± 20.2	− 0.32	− 3.46	[− 9.87; − 2.69]	< 0.001*
*Age group 50–59 (n* = *76)*						
BRI	40.6 ± 8.3	44.8 ± 9.4	*− 0.50*	− 4.40	[− 6.11; − 2.30]	< 0.001*
MI	54.9 ± 11.1	58.1 ± 11.4	− 0.29	− 2.53	[− 5.74; − 0.68]	0.014
GEC	95.5 ± 18.4	102.9 ± 19.4	− 0.41	− 3.54	[− 11.65; − 3.25]	0.001*
Informant report						
*Total group (n* = *169)*						
BRI	40.5 ± 8.8	44.5 ± 11.6	*− 0.46*	− 5.97	[− 5.41; − 2.72]	< 0.001*
MI	53.9 ± 13.0	59.7 ± 15.9	*− 0.44*	− 5.75	[− 7.69; − 3.76]	< 0.001*
GEC	94.4 ± 20.2	104.2 ± 25.6	*− 0.49*	− 6.35	[− 12.83; − 6.74]	< 0.001*
*Age group 40–49 (n* = *100)*						
BRI	40.2 ± 9.4	43.2 ± 10.7	− 0.32	− 3.23	[− 4.88; − 1.16]	0.002
MI	54.1 ± 13.2	58.0 ± 14.4	− 0.30	− 2.98	[− 6.54; − 1.31]	0.004
GEC	94.3 ± 20.8	101.2 ± 23.1	− 0.33	− 3.34	[− 11.06; − 2.82]	< 0.001*
*Age group 50–59 (n* = *66)*						
BRI	40.8 ± 7.8	45.5 ± 12.5	*− 0.61*	− 4.93	[− 6.63; − 2.81]	< 0.001*
MI	53.9 ± 12.9	60.1 ± 17.3	*− 0.48*	− 3.92	[− 9.36; − 3.04]	< 0.001*
GEC	94.7 ± 19.0	105.6 ± 28.0	*− 0.57*	− 4.66	[− 15.60; − 6.23]	< 0.001*

Data are presented as mean ± standard deviation. One sample *t* test was used to test the differences between CHD cohort and the norm in the self and informant reports. Norm data are derived from the Dutch/Flemish population aged 18–65 [26]. Four patients aged 60–64 were not compared with the mean of their age group seen the small sample size

*BRI* Behavioral Rating Index, *MI* Metacognition Index, *GEC* global executive composite

*Significant after Bonferroni correction for 36 comparisons

When analyzing per age group, CHD patients aged between 40 and 49 years scored significantly lower (better) than the norm population on all indexes for both self (BRI: *t* = − 4.05, *p* < 0.001, MI: *t* = − 2.58, *p* = 0.011, GEC: *t* = − 3.46, *p* < 0.001) and informant reports(BRI: *t* = − 3.23, *p* = 0.002, MI: *t* = − 2.98, *p* = 0.004, GEC: *t* = − 3.34, *p* < 0.001). However, after Bonferroni correction for 36 comparisons, the self-reported MI and informant-reported BRI and MI were no longer significant.

Older patients aged between 50 and 59, scored significantly better than the norm population on all indexes for both self (BRI: *t* = − 4.40, *p* < 0.001, MI: *t* = − 2.53, *p* = 0.014, GEC: *t* = − 3.54, *p* = 0.001) and informant reports (BRI: *t* = − 4.93, *p* < 0.001, MI: *t* = − 3.92, *p* = 0.011, GEC: *t* = − 4.66, *p* < 0.001). The self-reported MI was no longer significant after Bonferroni correction for 36 comparisons.

### Executive function per gender

**Fig. 2:**
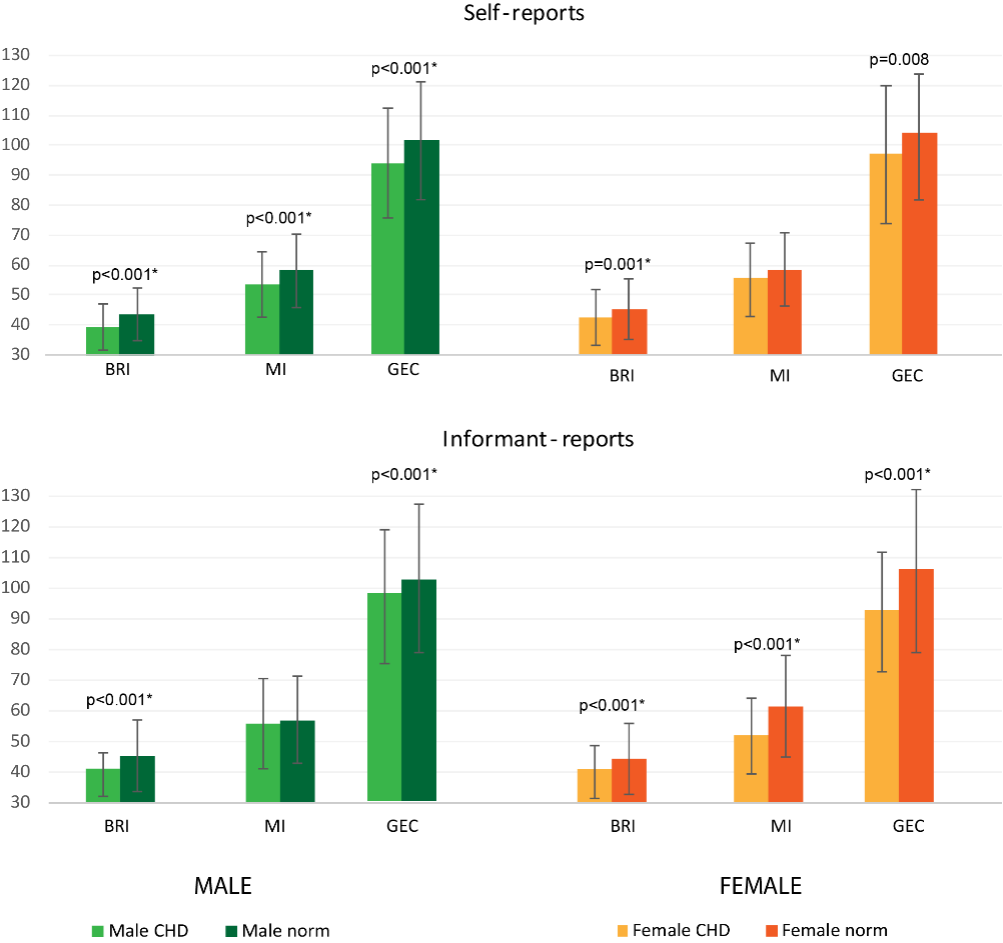
Brief-A self and informant reports raw scores: mean and standard deviation per gender in CHD group and norm (*n* = 1600 for the self-reports, *n* = 1082 for the informant reports). Norm data are derived from the Dutch/Flemish population from the manual [26]. T-scores were not available for normative data per gender. Error bar represents standard deviation. Higher scores indicate worse executive functioning. Only significant *p* values are reported. *Significant *p* values after Bonferroni correction per 36 correlations

Males with CHD scored significantly better on all the scales as compared to male norm population on the self-reports. Informants reported significantly better BRI and GEC scores if compared to the norm (Fig. 2).

Females with CHD scored significantly better on the BRI and GEC as compared to norm data. However, after Bonferroni correction, this difference was no longer significant on the GEC. Informants scored significantly better on both the indexes and GEC. These differences remained significant after Bonferroni correction for 36 comparisons.

Percentages of patients scoring in the clinical range are presented in Fig. 1S in the supplementary material.

### Mild and moderate/severe CHD

No significant differences on self and informant-reported EF were found between mild and moderate/severe CHD (Table 3S, supplementary material).

### Medical predictors for self-reported EF

The univariable analyses (Table 3) showed that female patients (*B* = 5.54, 95% CI [0.22; 10.85], *p* = 0.041), patients who take psychiatric medication (*B* = 14.17, 95% CI [5.08; 22.55], *p* = 0.001) and/or cardiac medication (*B* = 6.38, 95% CI [1.01; 11.75], *p* = 0.020) reported higher scores on the GEC. Patients with at least secondary (*B* = − 6.22, 95% CI [− 12.65; 0.22], *p* = 0.023) or higher (*B* = − 9.88; 95% CI [− 16.22; − 3.54], *p* = 0.002) education, with better exercise capacity (*B* = − 0.15, 95% CI [− 0.29; − 0.03], *p* = 0.013) with NYHA functional class 1 (*B* = − 12.36, 95% CI [− 20.15; − 4.57], *p* = 0.002) reported lower scores on the GEC.

**Table 3:** Prediction of outcome for total raw scores on the self- and informant-reports

	Self-total score Brief-A						Informant total score Brief-A					
	Univariate			Multivariate			Univariate			Multivariate		
	*B*	95% CI	*p*	*B*	95% CI	*p*	*B*	95% CI	*p*	*B*	95% CI	*p*
Age (years)	− 0.02	[− 0.55; 0.50]	0.927	–	–	–	− 0.27	[− 0.89; 0.35]	0.390	–	–	–
Gender (reference: male)	5.54	[0.22; 10.85]	0.041	–	–	–	− 4.81	[− 10.88; 1.26]	0.120	–	–	–
*Cardiac diagnosis (reference category: ASD)*												
VSD	− 1.03	[− 8.35; 6.29]	0.782	–	–	–	5.81	[− 2.62; 14.23]	0.175	–	–	–
PS	− 2.61	[− 11.53; 6.31]	0.564	–	–	–	− 0.02	[− 9.97; 9.93]	0.997	–	–	–
ToF	− 1.28	[− 8.91; 6.35]	0.742	–	–	–	1.15	[− 7.50; 9.81]	0.793	–	–	–
TGA	− 4.37	[− 14.15; 5.42]	0.380	–	–	–	7.08	[− 3.92; 18.07]	0.206	–	–	–
*Education level (reference: low)*												
Secondary	− 6.22	[− 12.65; 0.22]	0.023	–	–	–	− 0.74	[− 8.13; 6.66]	0.844	–	–	–
High	− 9.88	[− 16.22; − 3.54]	0.002	–	–	–	− 6.52	[− 13.98; 0.94]	0.086	–	–	–
Duration of pregnancy	0.21	[− 1.02; 1.43]	0.741	–	–	–	0.47	[− 0.96; 1.90]	0.521	–	–	–
Weight at birth	0.00	[− 0.01; 0.00]	0.244	–	–	–	0.00	[− 0.01; 0.00]	0.801	–	–	–
Palliative surgery before operation	1.35	[− 5.53; 8.24]	0.698	–	–	–	4.40	[− 3.37; 12.17]	0.265	–	–	–
Saturation before operation	0.25	[− 0.02; 0.52]	0.069	–	–	–	0.15	[− 0.15; 0.45]	0.320	–	–	–
Age at first heart operation	0.03	[− 0.71; 0.77]	0.935	–	–	–	− 0.45	[− 1.33; 0.42]	0.306	–	–	–
Aorta clamp	− 0.39	[− 11.45; 10.68]	0.945	–	–	–	11.12	[− 0.84; 23.08]	0.068	–	–	–
Time aorta clamp	− 0.05	[− 0.23; 0.12]	0.556	–	–	–	0.05	[− 0.17; 0.27]	0.662	–	–	–
Re-intervention with CPB	1.07	[− 5.96; 8.11]	0.764	–	–	–	1.86	[− 5.93; 9.93]	0.639	–	–	–
CVA and TIA	9.56	[− 3.09; 22.21]	0.138	–	–	–	11.77	[− 3.45; 27.00]	0.129	–	–	–
Heart failure	1.63	[− 12.71; 15.98]	0.823	–	–	–	1.93	[− 13.39; 17.26]	0.804	–	–	–
NT-pro-BNP	0.014	[− 0.12–0.14]	0.834	–	–	–	0.12	[− 0.02–0.26]	0.084	–	–	–
NYHA class 1	− 12.36	[− 20.15; − 4.57]	0.002	-	-	-	− 4.94	[− 14.21; 4.32]	0.294	-	-	-
Psychiatric medications	14.17	[5.08; 22.55]	0.001	12.51	[2.84; 22.18]	0.012	6.65	[− 2.74; 16.06]	0.164	–	–	–
Cardiac medications	6.38	[1.01; 11.75]	0.020	–	–	–	6.10	[− 0.09; 12.26]	0.053	–	–	–
Exercise test	− 0.15	[− 0.29; − 0.03]	0.013	− 0.15	[− 0.28; − 0.02]	0.021	− 0.25	[− 0.39; − 0.12]	< 0.001	− 0.27	[− 0.42; − 0.12]	< 0.001
*Systemic function (reference: good)*												
Reasonable	1.29	[− 21.09; 23.66]	0.910	–	–	–	− 4.07	[− 27.76; 19.62]	0.735	–	–	–
Poor	− 2.42	[− 11.91; 7.06]	0.615	–	–	–	5.89	[− 4.51; 16.28]	0.265	–	–	–
Bad	− 2.09	[− 24.01; 19.83]	0.851	–	–	–	4.08	[− 19.14; 27.31]	0.729	–	–	–

For the multivariate model, only the final model is presented. A broader significance selection *p* value (*p* < 0.2) was used to select the significant variables to build the multivariate model. *B* represents mean total scores changes on the self or informant reports

*ASD* atrial septal defect, *VSD* ventricular septal defect, *PS* pulmonary stenosis, *ToF* tetralogy of Fallot, *TGA* transposition of the great arteries, *CPB* cardiopulmonary bypass, *CVA* cerebrovascular accident, *TIA* transient ischemic attack

Multivariable analysis for self-reported executive functioning indicated that better exercise capacity was associated with better EF (*B* = − 0.15, 95% CI [− 0.28; − 0.02], *p* = 0.021), whereas taking psychiatric medication was associated with worse outcomes (*B* = 12.51, 95% CI [2.84; 22.18], *p* = 0.012).

### Medical predictors for informant-reported EF

The univariable analyses (Table 3) showed that patients with better exercise capacity (*B* = − 0.25, 95% CI [− 0.39; − 0.12], *p* < 0.001) obtained lower informant-reported scores on the GEC.

The multivariate analysis indicated that better exercise capacity (*B* = − 0.27, 95% CI [− 0.42; − 0.12], *p* < 0.001) was associated with better informant-reported executive functioning.

## Discussion

Our study focused on self- and informant-reported EF in 194 adults with CHD (median age of 50 years old) without any associated syndrome. In our study, we found that adults with CHD reported similar EF scores compared to healthy peers. In addition, no difference between diagnostic CHD groups was reported. The comparison per gender showed that both male and female patients scored better than the norm group. This result was unexpected since previous studies have shown that patients with CHD who underwent open-heart surgery are at higher risk of neurodevelopmental impairment [3].

Previous studies investigating neurocognitive outcomes in adults with CHD generally showed more impairment in terms of EF in CHD adults when compared to a control group. Furthermore, most of them confirmed this impairment also when comparing the CHD population to the norm, whereas only two studies showed no difference in term of EF between the CHD cohort and the norm population [11–15, 28–30]. However, all these past studies had a smaller sample size and generally focused on younger patients. In addition, they focused also on different types of CHD which we did not include in our study. Only a limited number of them focused exclusively on EF. Moreover, EF was evaluated through different types of tests, mostly performance-based assessments. In fact, two different types of tests can be used to assess EF: rating measures and performance-based tests. In the rating measures, like the BRIEF-A, patients are asked to estimate their daily performance in different situations engaging EF. Differently, the performance-based tests assess EF under optimal conditions, and they are interpreted by an external examiner. These two different types of tests assess different aspects of EF, so the measurements are not strongly associated and it is difficult to make direct comparisons between them [31].

If we focus on the studies which used BRIEF-A, results are contrasting: on the one hand, studies agree that there is no significant difference in term of EF between the CHD group and the norm population; on the other hand, two of them underlined the difference between the CHD and the control group [11–13, 15]. This could have been due to the fact that the control group size was relatively small, even smaller than the case group. Therefore, the control group may be a hyper functioning group not fully representative of the general population.

We found no difference between different diagnostic groups in term of EF. In the literature, results are contrasting. Some studies showed no significant difference based on the CHD severity [11, 13]. However, other studies found more neurocognitive impairment in severe CHD [12, 14]. This finding could be related to differences in inclusion of CHD diagnosis between studies. In fact, every CHD diagnosis may have different hemodynamic consequences, which can result in different effects on the neurocognitive system and a different impact on the EF. In fact, some of the previous studies included patients with more severe CHD, such as patients with Fontan circulation which could explain the worse outcomes [12, 28]. A previous study focusing on younger CHD population showed that more severe CHD are associated with worse EF [8].

Our study focuses on late adulthood with median age of 50 years old (range 40–66). This age may represent the ideal timing to measure EF. In fact, while older patients may have experienced age-related dementia, younger patients may have underlying conditions which were not yet investigated. Furthermore, better scores of the CHD patients could be related to the fact that these were the first operations of this type in Rotterdam. Therefore, a selection of suitable patients for the surgery could have occurred. In addition, our study excluded all the syndromic patients and/or patients with moderate to severe psychomotor deficits.

Generally, higher education was related to better scores on self-reports in the univariate analysis. This finding is supported by previous studies which have shown that higher education is related to better cognitive capacities also in older adults [32].

The univariable analysis indicated that better EF outcomes (self-reports) were related to better exercise testing performance and/or better functional capacity (NYHA class 1), whereas taking cardiac medication was related to worse EF. All these factors relate to a worse cardiac health, therefore a limited involvement in exercise activities. Worse exercise testing performance is generally also associated with a sedentary lifestyle, whereas studies largely showed the benefits of physical activity on EF [33, 34]. A direct association between cardio-respiratory fitness (CRF) and cognitive functioning was also shown. In fact, patients with significant improvements in their CRF reported also significant improvements in multiple cognitive domains, included EF [34].

Patients using psychiatric medications scored worse on the self-reports. This could be related to an already existing psychiatric condition that may be associated with limited executive functions or to side effects of the medication themselves. For instance, patients who are currently depressed or recently remitted show impairment in cognitive function as well in the EF domains as in memory and attention [35, 36]. Likewise, similar findings are documented for attention-deficit hyperactivity disorder. Nonetheless, the level of psychopathology in our cohort was similar to adults from the normal population. However, female patients had higher levels of somatic complaints [37].

### Strengths and limitations

Our study included a relatively large number of CHD adults (*n* = 194) and it studied patients with CHD in their middle adulthood, while other studies included a smaller sample size and younger patients. This group of patients was followed clinically in our hospital for over 40 years. Internationally standardized questionnaires were used to evaluate the EF of our cohort.

It must be also considered that the response rate was 45% of the eligible cohort of patients. This may have biased our cohort with higher participation of patients with a higher education level and a better health condition. However, no significant difference was found in terms of medical history and biological characteristics between participants and non-participants.

This study took into consideration only five CHD diagnostic groups (ASD, VSD, PS, ToF, TGA), operated a long time ago; therefore, caution is appropriate with regard to other diagnostic groups and results after contemporary surgery. Particularly, the lack of patients with single ventricular heart defects treated with a Fontan circulation may be considered a weakness of this study. The concept of this approach was developed ± 50 years ago, so very few patients have reached the age of our current cohort [38].

## Conclusion

This study showed favorable reassuring findings in terms of EF for adults with CHD.

Our cohort of non-syndromic adults with CHD did not show worse outcomes compared to the general population. In the final multivariable regression model, taking psychiatric medication predicted worse outcomes for self-reported EF, while a better performance at the exercise test was linked to better self- and informant-reported EF.

These promising results are of great importance not only for adults with CHD, but also to newborns and future parents of CHD children, who could achieve not only long-term survival, but also good long-term quality of life.

### Supplementary Information

Below is the link to the electronic supplementary material.

Supplementary file1 (DOCX 81 KB)

## Data Availability

The anonymous data that support the findings of this study are available from the author, upon reasonable request.
