# Supplementary material for: Executive functioning of patients with congenital heart disease: 45 years after surgery
Source: Clin Res Cardiol. 2023 Apr 9;112(10):1417–26. doi: 10.1007/s00392-023-02187-3 (PMC10562274; doi:10.1007/s00392-023-02187-3)
Supplement: Supplementary file 1 — Supplementary file1 (DOCX 81 KB) [file 392_2023_2187_MOESM1_ESM.docx]

**Supplementary material:**

**Table1S.** Patients flowchart

Original cohort of CHD patients operated between 1968-1980

(n=597)

Participants:

(n=194)

Invited patients:

(patients who participated to at least two of the previous follow-up times and were traceable)

(n=343)

Alive patients and survival status available at the 4^th^ follow-up time

(n=431)

All the alive patients who did not participate to the study were defined as non-participants (n=237)

**Table 2S**. Differences between participants and non-participants of the original cohort of operated patients. (431)

|  | **Non-participants**  **Alive***  **(n= 237)** | **Participants**  **(n=194)** | **p** |
| --- | --- | --- | --- |
| **VARIABLE** | | | |
| *Biological characteristics* | | | |
| Moderate/severe CHD | 46.4% (110) | 45.9% (197) | 0.911 |
| Female | 48.1% (114) | 45.9% (89) | 0.645 |
| *Medical history* | | | |
| Duration of pregnancy (weeks) | 40.0 [40.0-40.0] | 40.0 [40.0-40.0] | 0.061 |
| Weight at birth (kg) | 3.2 [2.8-3.6] | 3.2 [2.8-3.6] | 0.635 |
| Palliative surgery before the surgical repair | 24.1% (57) | 18.6% (36) | 0.168 |
| Saturation before operation | 94.0 [82.0-97.0] | 94.0 [82.5-97.7] | 0.583 |
| Age at first open heart operation (years) | 4.5 [0.9-7.2] | 4.8 [1.3-7.2] | 0.659 |
| Clamp of the aorta | 95.1% (215) | 93.1% (163) | 0.395 |
| Time aorta clamp (minutes) | 40.0 [21.0-54.0] | 39.0 [19.7-52.2] | 0.456 |

*Information regarding survival status in 2021 were available for 93% of the original operated cohort (597 patients).

For continuous variables median [25^th^-75^th^ percentile] are reported. For categorical variables, percentage (n) are shown. Differences between diagnostic groups were analyzed for the continuous data with Mann-Whitney-U test since not normally distributed. Chi^2^-test was used to analyze differences between categorical data.

**Table 3S**. Brief A self-report and informant-report raw scores per CHD group.

|  | **Mild CHD**  **(n=131)** | **Moderate/severe CHD**  **(n=63)** | **P** |
| --- | --- | --- | --- |
| **Self-report** |  |  |  |
| BRI | 40.9 ± 8.9 | 40.0 ± 7.7 | 0.702 |
| MI | 54.5 ± 11.9 | 54.3 ± 11.1 | 0.973 |
| GEC | 95.4 ± 19.7 | 94.1 ± 17.1 | 0.904 |
| **Informant-reports** | |  |  |
| BRI | 40.7 ± 9.0 | 39.9 ± 8.5 | 0.597 |
| MI | 53.4 ± 13.0 | 55.1 ± 12.9 | 0.336 |
| GEC | 94.1 ± 20.4 | 95.0 ± 19.5 | 0.692 |

BRI: Behavioral Rating Index, MI: Metacognition Index, GEC: Global Executive Composite

Data are presented as mean ± standard deviation. Mann-Whitney-U test was used to test the difference between CHD groups respectively for self and informant-reports.

**Figure 1S.** Percentage of patients scoring in the clinical range.


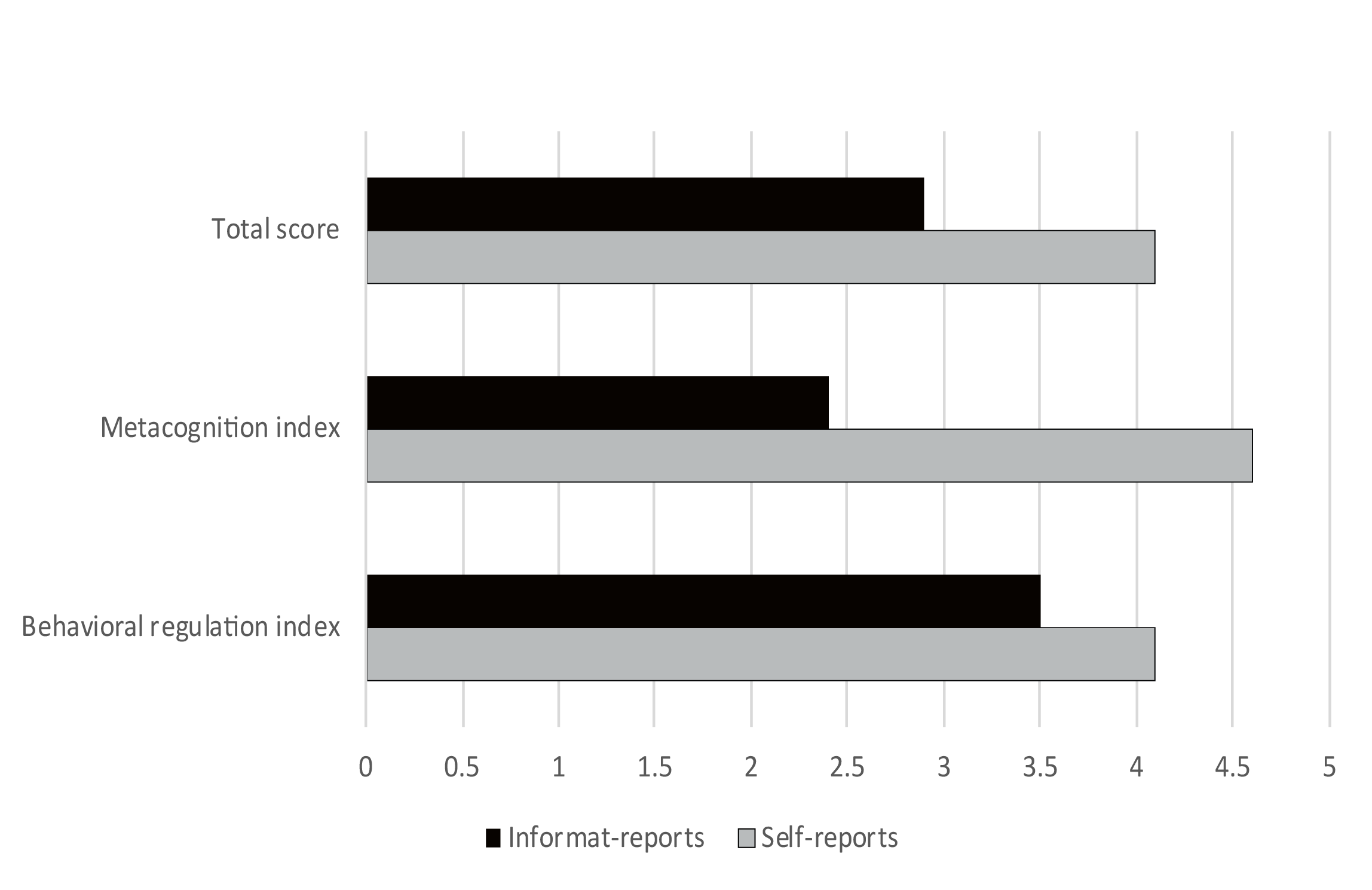


Clinical range was defined as 1.5 SD from the normative mean. [1, 2]

**References:**

1. Gioia GA, I.P., Guy SC, *BRIEF: Behavior Rating Inventory of Executive Function*. 2000, Lutz, FL: Psychological Assessment Resources.

2. Scholte, E., & Noens, Ilse, *BRIEF-A. Vragenlijst over executieve functies bij volwassenen.* 2011, Amsterdam: Hogrefe Uitgevers B.V.
